# Association of serum concentrations of perfluoroalkyl compounds with poor growth and failure to weight gain in 2-year-old children

**DOI:** 10.1186/1687-9856-2015-S1-P31

**Published:** 2015-04-28

**Authors:** Young Ah Lee, Jin Hee Kim, Hwa Young Kim, Haewoon Jung, Jieun Lee, Juyoung Yoon, Sanghyuk Bae, Yun-Chul Hong, Choong Ho Shin, Sei Won Yang

**Affiliations:** 1 Department of Pediatrics, Seoul National University Children's Hospital, Seoul, Korea; 2 Environmental Health Center, Seoul National University College of Medicine, Seoul, Korea; 3 Department of Environmental Health, Graduate School of Public Health, Seoul Nati, Korea; 4 Institute of Environmental Medicine, Seoul National University Medical Research, Seoul, Korea

## Backgrounds

Potential health concerns of perfluoroalkyl compounds (PFCs) have been raised.

## Objectives

We investigated the relationship between exposure to PFCs and growth parameters in Korean 2-year-old children.

## Methods

Three hundred sixty children (189 boys, 1.9 to 2.2 years) born as appropriate gestational age infants were enrolled. Height and weight at visit, birth weight, midparental height (MPH) and bone age (BA) were evaluated.

## Results

Among fifteen PFCs analyzed, perfluorohexane sulfonic acid (PFHxS), and perfluorooctane sulfonic acid (PFOS), perfluorooctanoic acid (PFOA), perfluorononanoic acid (PFNA), and perfluorodecanoic acid (PFDA) were detected in >90% of the serum samples. The number of chemicals above median concentrations among these 5 PFCs were graded on a scale of 0 to 5, and classified into exposure (0) (n = 97), exposure (1-2) (n = 88), and exposure (≥3) groups. After adjusting for sex, birth weight, MPH, and BA, log-transformed PFHxS, PFOS, PFOA, and PFDA were associated with a 1.60, 1.35, 1.57, 1.29 cm decrease in height (all P < 0.005). Log-transformed PFOS, PFOA, PFNA, and PFDA were negatively related to weight gain (all P < 0.05). Change in weight Z-scores decreased progressively from exposure (0), to exposure (1-2), and to exposure (≥3) (mean +0.43 vs. +0.29 vs. +0.10, P = 0.012).

## Conclusions

Increased concentrations of PFOS, PFOA, PFNA, and PFDA were associated with short stature and failure to weight gain in 2-year-old children. The more PFCs detected above median concentrations, the shorter and the poorer weight gain. Further prospective studies are needed to clarify causal relationship.

